# Clinical efficacy of short-term prewarming in elderly and adult patients: A prospective observational study

**DOI:** 10.7150/ijms.77578

**Published:** 2022-09-06

**Authors:** Sung-Ae Cho, Sieun Yoon, Seok-Jin Lee, Young-Seok Jee, Choon-Kyu Cho, Tae-Yun Sung

**Affiliations:** 1 Department of Anaesthesiology and Pain medicine, Konyang University Hospital, Myunggok Medical Research Center, Konyang University College of Medicine, Daejeon, Korea.; 2 Department of Anaesthesiology and Pain medicine, Konyang University Hospital, Konyang University College of Medicine, Daejeon, Korea.

**Keywords:** Hypothermia, Geriatrics, Incidence, Perioperative care

## Abstract

**Background:** Short-term prewarming effectively reduces intraoperative hypothermia in adult patients. However, few data exist regarding its efficacy in elderly patients. Elderly people have a reduced ability to regulate their body temperature, which affects the efficacy of prewarming. This study aimed to compare the clinical efficacy of short-term pre-warming in elderly patients with that in adult patients.

**Methods:** We enrolled 25 adult (20-50 years) and 25 elderly (> 65 years) patients scheduled for ureteroscopic stone surgery under general anaesthesia. All patients received preanaesthetic forced-air warming for 20 min. The core temperature was measured using an infrared tympanic thermometer during awakening and nasopharyngeal thermistors during anaesthesia. Incidence and severity of intraoperative hypothermia (< 36°C) was compared. Postoperative shivering and number of patients requiring active warming in the post-anaesthesia care unit were also assessed.

**Results:** Intraoperative hypothermia was more frequent in elderly than in adult patients (58.3% *vs*. 12.0%; relative risk 2.6; 95% confidence interval 1.5 to 4.6; effect size *h* = 1.010; *p* = 0.001). The severity of intraoperative hypothermia showed a significant intergroup difference (*p* = 0.002). Postoperative shivering was more frequent in elderly than in adult patients (33.3% *vs*. 8.0%, *p* = 0.037). A greater number of elderly patients in the post-anaesthesia care unit required active warming (33.3% *vs*. 8.0%, *p* = 0.037).

**Conclusions:** The effects of short-term prewarming on the prevention of hypothermia and maintenance of perioperative normothermia are not the same in the elderly and adult patients.

## Introduction

Inadvertent intraoperative hypothermia (core temperature < 36.0°C) is associated with perioperative adverse events, such as an increase in morbid myocardial outcome, wound infections, coagulopathy, blood loss and transfusion requirements, postoperative shivering, discomfort, and prolonged hospital stay 1-3. Elderly people are vulnerable to hypothermia owing to their decreased ability to regulate body temperature through vasoconstriction, shivering, and reduction in muscle mass, fat, and subcutaneous tissues 4,5. Furthermore, their reduced physiological reserves increase the risk of hypothermia-related adverse events 5. Therefore, prevention of inadvertent hypothermia should be mandatory in elderly patients undergoing elective surgery 6.

The redistribution of body heat from the core (e.g. thorax and abdomen) to the periphery (e.g. legs, arms) due to anaesthetic-induced impairment of thermoregulation is the primary cause of hypothermia in patients administered general anaesthesia and accounts for 81% of the decrease in core temperature during the first hour 1,2,7,8. Even if active warming is applied during surgery, redistribution hypothermia is difficult to treat because the core-to-peripheral flow of heat is massive, and it takes a considerable amount of time for the applied cutaneous heat to be transferred to the core tissues 2. However, it can be prevented by active warming of the skin surface before the induction of anaesthesia (i.e. prewarming) 3. Prewarming does not significantly increase core temperature due to normal thermoregulation, such as vasodilation and sweating, but reduces the temperature gradient between the core and periphery, thereby reducing the initial drop in core temperature 1,6,7.

Prewarming is recommended for a minimum of 30 min (up to 60 min) 7, but this is impractical in a busy operating room environment. In several subsequent studies 9,10, short-term prewarming for 10-30 min also effectively reduced the incidence of hypothermia in adult patients compared to the control group. Short-term prewarming is recommended to prevent hypothermia in clinical situations where prewarming for > 30 min is not easy 1,3. However, to our knowledge, the efficacy of short-term prewarming in elderly patients vulnerable to inadvertent hypothermia has not been established. Therefore, we designed this study to compare the efficacy of short-term prewarming for 20 min in elderly patients with that in adult patients.

## Materials and Methods

### Ethics and population

This prospective observational study was approved by the Institutional Review Board of Konyang University Hospital, Daejeon, Republic of Korea (approval number KYUH 2020-12-003-001 n 30 December, 2020; Chairperson Prof. JW Son), and the protocol was registered with the Korean Clinical Research Information Service (https://cris.nih.go.kr/, permit number: KCT0005781). This study adhered to the Strengthening the Reporting of Observational Studies in Epidemiology (STROBE) guidelines. This prospective, non-randomised, comparative study was conducted between February 2021 and December 2021 at a single university hospital after obtaining written informed consent from participants and/or legal surrogates.

In this study, we included consecutive patients aged 20-50 years (adult group) and > 65 years (elderly group), with American Society of Anesthesiologists physical status (ASA PS) I to II, scheduled for elective ureteroscopic stone surgery under general anaesthesia, and expected duration of anaesthesia > 30 min. The exclusion criteria were preoperative core temperature ≥ 37.6°C or < 36.0°C; body mass index (BMI) > 35 kg/m^2^; severe endocrine, cardiovascular, or respiratory disease; and cognitive impairment or a neuropsychological disorder.

### Study protocol

An identical study protocol was applied to all participants, as follows: all participants fasted for at least 8 h and did not receive premedication. At our hospital, the ambient temperature of the pre-anaesthesia holding area and post-anaesthesia care unit (PACU) was maintained at 22-25°C. In the pre-anaesthesia holding area, all participants received 20 min of active warming using a forced-air blanket (Bair Hugger^TM^ Full Body Blanket Model 30000, Arizant Healthcare Inc., Eden Prairie, MN, USA), which was placed over the entire body and then covered with a cotton blanket. During the active warming period, the forced-air warming device (Bair Hugger^TM^ Model 505 Warming System, Arizant Healthcare Inc., St. Paul, MN, USA) was set to 43°C (“high”), but if patients complained that it was too warm, the warming temperature was reduced to 38°C (“medium”). After 20 min of warming, the forced-air warming device was turned off and all participants were transferred to the operating room with a cotton blanket over a force-air blanket.

Upon arrival to the operating room, patients underwent anaesthetic monitoring, including pulse oximetry, electrocardiography, non-invasive automated blood pressure, patient state index (PSI; SedLine®; Masimo Corp., Irvine, CA, USA), and neuromuscular train-of-four by acceleromyography. General anaesthesia was induced with intravenous propofol (1.5-2 mg/kg) and fentanyl (1-2 μg/kg), and endotracheal intubation was facilitated with rocuronium (0.6 mg/kg). After anaesthesia induction, the posture of all patients was changed to the lithotomy position for surgery. During surgical preparation, including surgical scrubbing and draping, a forced-air warming blanket was placed on the upper body above the xiphoid process, including the patient's arms. At the end of surgical preparation, active warming with a forced-air warming device was restarted, and the set temperature of the warming device was maintained at 38°C throughout the surgery.

Anaesthesia was maintained with an oxygen/nitrous oxide mixture (50:50) and desflurane, and the end-tidal concentration of desflurane was adjusted to maintain PSI at 25-50. During anaesthesia, inhaled gas was supplied through a heated (39.5°C) and humidified respiratory circuit. All intravenous fluids used for anaesthesia and irrigation were administered at room temperature. Hypotension (systolic blood pressure < 80% of pre-induction systolic blood pressure or mean blood pressure < 60 mmHg) and hypertension (systolic blood pressure > 120% of pre-induction systolic blood pressure or systolic blood pressure > 180 mmHg) were treated with 50 μg of intravenous phenylephrine and 0.5 mg of nicardipine, respectively. In cases of persistent (> 2 min) bradycardia (heart rate < 50 beats/min) and tachycardia (heart rate > 120 beats/min), 0.2 mg of glycopyrrolate and esmolol (10 mg) were administered intravenously. Ephedrine (5 mg) was administered intravenously if the hypotension was accompanied by bradycardia. After surgery, the patients received sugammadex to antagonise neuromuscular block and were transferred to the PACU.

In the PACU, active warming using a forced-air warmer set to 43°C was applied when patients complained that they were feeling cold or shivering or if their temperature was less than 36°C.

### Measurements and outcomes

All perioperative outcomes were evaluated by an anaesthesiologist who was blinded to the study.

In the awake state, that is, before induction of anaesthesia and after completion of general anaesthesia, the patient's temperature was measured using an infrared tympanic thermometer (Thermoscan IRT 4020, Braun GmbH, Kronberg, Germany; accurate to ± 0.2°C for patient temperatures in the range of 35.5-42°C and to ± 0.3°C for patient temperatures < 35.5°C) by a trained anaesthesiology resident. The anaesthesiology resident selected one ear without excessive earwax or obstructions after examining both ears through otoscopy prior to measuring tympanic temperature. Tympanic temperature was measured using a thermometer according to the manufacturer's instructions. The highest temperature in the same ear was recorded after at least three consecutive measurements. In contrast, immediately after induction of general anaesthesia to extubation, the patient's core temperature was measured in the nasopharynx by inserting a 12 Fr thermistor probe (L000412, Gonimed Co., South Korea) at a depth of 10-20 cm from the nares 11. The patient's temperature was recorded on arrival in the pre-anaesthesia holding area (baseline), on arrival in the operating room, every 15 min immediately after induction of anaesthesia to the end of surgery, and every 15 min from PACU admission for 1 h by the same anaesthesiology resident. The ambient operating room temperature was measured using a thermistor (L000412, Gonimed Co., South Korea) located at the height of the operating table and away from the cooling or heating equipment. The ambient operating room temperature was recorded at the start and end of the surgery. After completion of anaesthesia, the amounts of intravenous and irrigation fluids measured by the anaesthesiology resident and blood loss by the surgeon were recorded.

Hypothermia was defined as a patient's tympanic or nasopharyngeal temperature < 36°C, and the severity of hypothermia was graded as mild (35-35.9°C), moderate (34-34.9°C), or severe (≤ 34°C) 12. Thermal comfort was assessed immediately after baseline temperature measurements in the pre-anaesthesia holding area and after arrival at the PACU using a numeric rating scale (0=completely uncomfortable, 10=completely comfortable) 13.

In the PACU, postoperative pain was evaluated using the numerical rating scale (NRS) (0=no pain; 10=worst possible pain) and intravenous fentanyl (0.5-1 μg/kg) was injected when NRS was ≥ 4.

Shivering was assessed using a four-point scale (0=no shivering; 1=intermittent, low-intensity; 2=moderate; 3=continuous and intense shivering) 9, and the highest score was recorded. A shivering score ≥ 1 was considered for the incidence of shivering. If the shivering scale score was ≥ 2, 25 mg meperidine was administered intravenously. Intravenous metoclopramide (10 mg) was administered for postoperative nausea and vomiting. Additionally, all adverse events that occurred during recovery in the PACU were documented.

The primary outcome was the incidence of intraoperative hypothermia. The secondary outcomes were the severity of intraoperative hypothermia, changes in temperature during the perioperative period, thermal comfort scores, incidence of shivering, and the number of patients requiring active warming in the PACU.

### Statistical analyses

Sample size was calculated using G*Power (version 3.1.9.7; Franz Faul, Universitat Kiel, Germany). In our preliminary study (n=12 in each group), the incidence of intraoperative hypothermia was 16.7% (2/12) in the adult group and 58.3% (7/12) in the elderly group. Based on the results of this preliminary study, a sample size of 21 patients per group was calculated, with an effect size *h* = 0.840, an α-value of 0.05 (two-tailed), a power of 0.8, and an allocation ratio of 1:1. Considering the potential dropout rate of 15%, 25 patients were enrolled in each group.

Statistical analyses were performed using the SPSS software (ver. 27 for Window; SPSS Inc., Chicago, IL, USA). After normality was checked with the Kolmogorov-Smirnov test, continuous variables were analysed using the Student's *t*-test or Mann-Whitney *U*-test and are presented as mean ± standard deviation or median (interquartile range). Repeatedly measured variables (temperature) were analysed using a linear mixed model with a Bonferroni correction. In this model, the fixed effects were the group, time, and the interaction between group and time, and the random effect was the subject. Categorical variables were analysed using the χ^2^ test, χ^2^ test for trends (linear-by-linear association), or Fisher's exact test, as appropriate, and are presented as a number or number (%). In all analyses, statistical significance was set at *P* < 0.05. Cohen's effect sizes *d* and *h* were used to compare the continuous and categorical variables, respectively.

## Results

**Figure 1:**
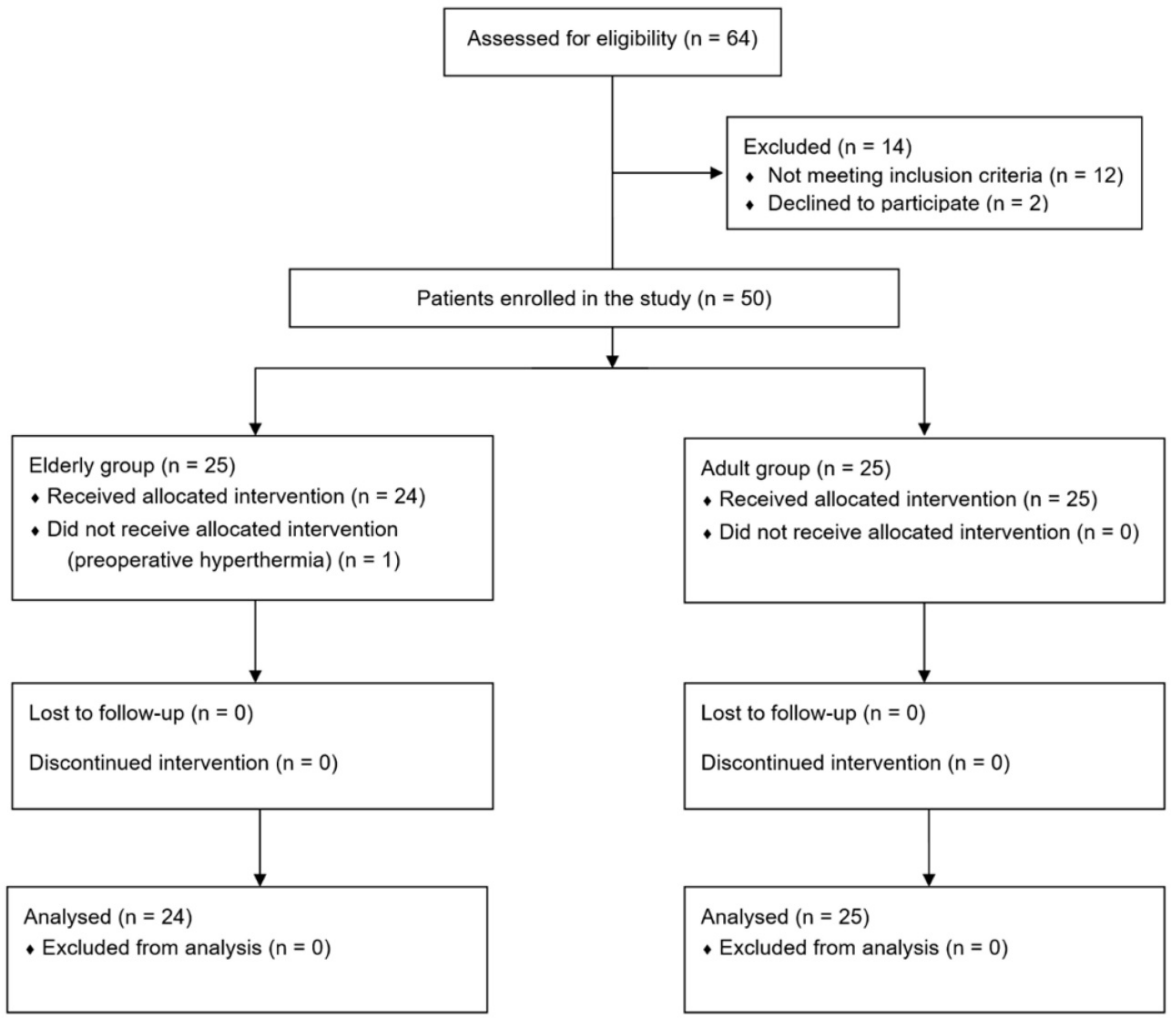
Flow chart.

Sixty-four patients were screened, 14 of whom were excluded and 25 were enrolled in each group. One patient enrolled in the elderly group had a baseline core temperature of 37.6°C on the day of surgery, did not receive prewarming, and was excluded from this study. Thus, 24 and 25 patients in the elderly and adult groups, respectively, completed the study (Fig. 1).

The patient characteristics and perioperative data are presented in Table 1. Patients' weight and height were lower in the elderly group than in the adult group (*P* = 0.004 and *P* < 0.001, respectively), but BMI did not show significant intergroup differences (*P* = 0.206). ASA PS was class II in the elderly group, whereas 68% (17/25) of the patients in the adult group were class II (*P* = 0.004). In the elderly group, all patients underwent prewarming with the forced-air warming device set at a temperature of 43°C; however, in the adult group, one patient complained that it was too warm during prewarming, so the device's set temperature was adjusted from 43°C to 38°C (*P* > 0.999). Time taken from the end of prewarming to the induction of anaesthesia was not different between the groups (*P* = 0.149), but the time taken from the end of prewarming to the resumption of forced-air warming during surgery was longer in the elderly group than in the adult group (22.1 ± 3.1 *vs*. 20.5 ± 2.6 min; mean difference [MD] 1.6 min; 95% confidence interval [CI] for MD 0.03 to 3.3 min; effect size *d*=0.559; *P* = 0.046). Sex, stone position, preoperative core temperature, preoperative thermal comfort score, operating room temperature, amount of intravenous and irrigation fluids, estimated blood loss, duration of surgery, and anaesthesia were not significantly different between the groups.

The perioperative outcomes are presented in Table 2. The incidence of intraoperative hypothermia (the primary outcome) was higher in the elderly group than in the adult group (58.3% [14/24] *vs*. 12.0% [3/25]; relative risk [RR] 2.6; 95% CI for RR 1.5 to 4.6; effect size *h*=1.010; *P* = 0.001). The severity of intraoperative hypothermia was significantly different between groups (*P* = 0.002). In the elderly group, 41.7% showed mild hypothermia and 17.7% showed moderate hypothermia; in the adult group, only mild hypothermia occurred in 12% of patients. In the PACU, the incidence of shivering was higher in the elderly group (33.3% [8/24]* vs*. 8.0% [2/25]; RR 2.0; 95% CI for RR 1.2 to 3.2; effect size *h*=0.657; *P* = 0.037). A score of 2 was not observed in either group. The number of patients requiring active warming was significantly higher in the elderly group than in the adult group (66.7% [16/24] *vs*. 32.2% [8/25]; MD: 34.7%; 95% CI: 6.7%-56.0%; effect size *h*=0.705; *P* = 0.015). There were no differences between the two groups in terms of the NRS pain score, number of patients receiving fentanyl, and postoperative thermal comfort score.

**Figure 2:**
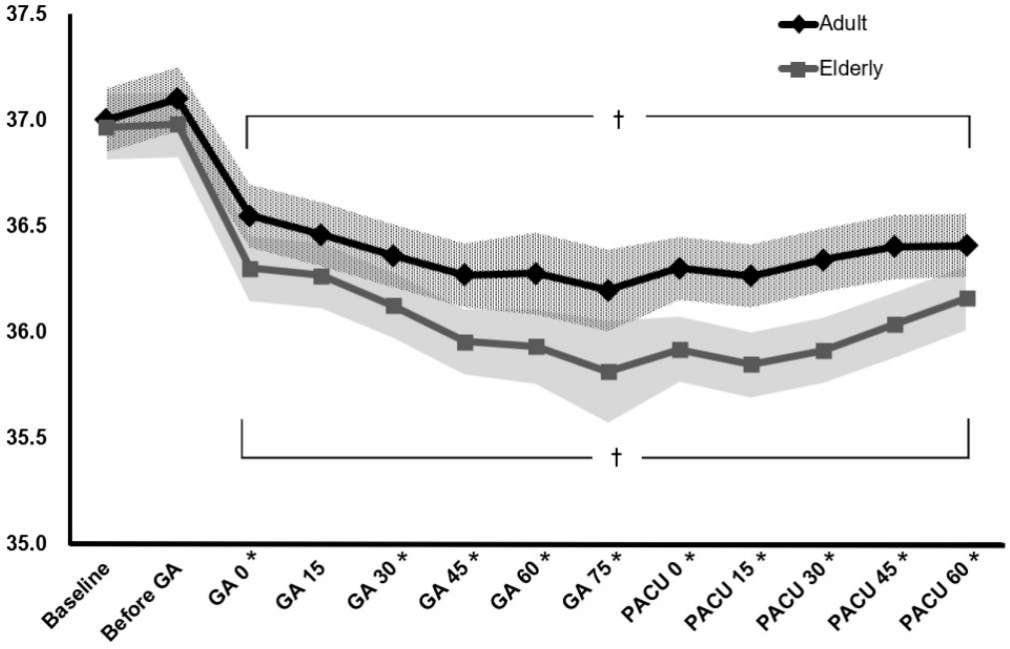
** Changes in perioperative core temperature.** The value of temperature is presented with estimated means and 95% confidence intervals according to a linear mixed model. Baseline: temperature at the arrival of the pre-operative holding area, Before GA: temperature on arrival in the operating room, GA0-GA60: temperature immediately to 60 min after induction of general anaesthesia (checked every 15 min), PACU0-PACU60: temperature immediately to 60 min after arrival of the post-anaesthetic care unit (checked every 15 min). ^*^p < 0.05, compared between the two groups (Bonferroni corrected). ^†^*p* < 0.05, compared to the baseline temperature in each group (Bonferroni corrected).

The changes in perioperative core temperature are illustrated in Fig. 2. The interaction between group and time was significant, suggesting that the trend of core temperature change was significantly different between the elderly and adult groups during the perioperative period (*P* < 0.001). In addition, the overall mean difference in perioperative temperature between the two groups was 0.3 °C (95% CI 0.1 to 0.5, *P* = 0.002). Temperatures measured immediately after the induction of general anaesthesia to 60 min after arrival at the PACU were significantly different from the baseline temperature within each group. In the post hoc analysis, core temperatures were lower in the elderly group than in the adult group from immediately after anaesthesia induction to 60 min after arrival at the PACU, except 15 min after anaesthesia induction (*P* = 0.076) (Table 3).

None of the participants had thermal injury due to forced air warming, and adverse events were comparable between the two groups (Table 4).

## Discussion

This study aimed to compare the clinical efficacy of short-term prewarming in elderly and adult patients. After the application of prewarming with a forced-air warming blanket for 20 min, intraoperative hypothermia and postoperative shivering were more common in elderly patients than in adult patients, and the severity of hypothermia was worse in elderly patients. The number of elderly patients requiring active warming in the PACU due to hypothermia, cold, or shivering was significantly higher. In addition, a marked difference in the trends of core temperature change during the perioperative period was observed between the elderly and adult patients. The findings of this study suggest that the efficacy of prewarming for the prevention of inadvertent hypothermia in elderly patients is inferior to that in adults and that additional strategies to prevent inadvertent hypothermia are needed in elderly patients.

Anaesthesia-induced redistribution of body heat is the primary cause of inadvertent intraoperative hypothermia 1,2, and the best way to prevent redistribution hypothermia is prewarming 14. Therefore, prewarming is recommended in almost all patients to prevent perioperative hypothermia, except for emergency surgery, which can be dangerous when surgery is delayed owing to prewarming 6. The factors to consider while applying prewarming with the forced-air warming device are the set temperature and duration of the device. Of the two factors, the set temperature of the forced-air warming device used during prewarming is considered as the highest temperature recommended by the manufacturer because there is no rationale for reducing the efficacy of forced-air warming by lowering the set temperature of the warmer below that recommended by the manufacturer 1. Despite the same duration of prewarming and similar surgical settings, prewarming in a high-temperature setting (45 °C) resulted in improved thermal benefits compared to a moderate temperature setting (38 °C) 15. This might be because prewarming in a high temperature setting increased the peripheral heat contents more than that in a moderate temperature setting 7. Meanwhile, the recommended duration of prewarming varies from 10 to 30 min 3 or ≥ 30 min 16, depending on the clinical guidelines related to the prevention of perioperative hypothermia. However, a randomised controlled study suggested that increasing the duration of prewarming beyond 30 min would not better preserve intraoperative normothermia 17. In addition, 20 min of prewarming showed no significant difference in the incidence of hypothermia at the end of anaesthesia compared to 30 min (7% vs. 6%, respectively) 9. As in our study, when intraoperative active warming was applied to patients, the effects of short-term prewarming for 5-15 min or 10 min showed different results depending on the studies 18,19. Patients prewarmed for 5-15 min showed significantly higher core temperature throughout the intraoperative period than non-prewarmed patients 18. In contrast, prewarming for 10 min did not further reduce the incidence of intraoperative and postoperative hypothermia in patients undergoing elective surgery of less than 120 min 19. Based on the results of these observations 1,9,17-19, the prewarming temperature was set to 43 °C, and the duration was set to 20 min in this study.

In addition to the set temperature of the warming device and duration of prewarming, factors affecting the efficacy of prewarming include sweating, thermal discomfort of the patient, ambient temperature of the place where prewarming was performed and the operating room, blanket properties (e.g. design and size), surface area covered with a blanket, duration of surgery, type of surgery, and interrupted time between the end of prewarming and the start of intraoperative warming 2,7,17. Prolonged warming (for > 1 h) may induce sweating, which may decrease the efficacy of prewarming, but in our study, the effect of sweating in short-term prewarming was negligible 7. The thermal discomfort during prewarming may cause cessation of prewarming or adjustment of thermostat ("lowering") of the warmer, thereby reducing the heat content delivered to the patient 7. In this study, one patient in the adult group complained of thermal discomfort during prewarming; however, no statistical difference was observed between the two groups in the number of patients requiring adjustment of the warmer thermostat during prewarming. In addition, the same blanket was applied to the patient's whole body in the same place in both groups, and there was no difference between the groups in terms of ambient operating room temperature, duration, and type of surgery.

The time from the end of prewarming to the resumption of intraoperative forced-air warming was longer in the elderly group than in the adult group. This is probably due to the specific conditions observed in elderly patients, such as frailty, limited mobility, and hearing impairment 20. It would have taken more time in elderly patients than in adult patients to move to the operating table after prewarming, identify the patient and the surgical site through open-ended questions before induction of anaesthesia, and switch to the surgical position following induction of anaesthesia. In a study conducted on patients aged 18-85 years who underwent non-cardiac surgery under general anaesthesia, the risk of intraoperative hypothermia increased by 4.9% for every minute of delay between the end of the prewarming and initiation of intraoperative warming 17. However, considering that the mean difference between the two groups in the warming interruption time in our study was only 1.6 min, this factor alone was insufficient to explain the difference (46.3%) in the incidence of intraoperative hypothermia between elderly and adult patients. The results of this study suggest that aging is also a factor that affects the efficacy of prewarming.

Despite the application of the same method of prewarming and intraoperative thermal manipulation, there are many reasons explaining how elderly people are more susceptible to intraoperative hypothermia. First, to reduce the temperature gradient between the core and periphery by prewarming, the process of vasodilation must be preceded 21, and increased perfusion to the peripheral compartments by vasodilation during prewarming results in an increase in heat content and a reduction in the temperature gradient for redistribution 14. However, venous stiffening with aging reduces the ability to buffer changes in blood distribution, and increased levels of circulating norepinephrine in elderly people lead to increased arteriole constriction and systemic vascular resistance 22. Therefore, we speculate that prefusion and heat transfer to the peripheral compartments by vasodilation during prewarming may be less than that in elderly people. Second, vasoconstriction and shivering are major autonomic thermoregulatory responses to cold in patients undergoing anaesthesia 8. However, in elderly patients, thermoregulation via vasoconstriction and shivering was less effective than that in younger patients, regardless of whether anaesthesia was present or not 23, and the decrease in the threshold of vasoconstriction by anaesthetics was more profound (approximately 1 °C) than that in younger patients 24. Third, the decrease in skeletal muscle mass and subcutaneous (insulating) fat reserves with age would have contributed to the high incidence of hypothermia in elderly patients by impairing their ability to produce and conserve heat 4,5.

Based on the results of this study, it seems necessary to apply a longer duration of prewarming in elderly patients than in adult patients or additional thermal management to expect a similar effect for hypothermia prevention. In elderly patients in a haemodynamically stable state, the administration of vasodilators before anaesthesia (i.e. pre-dilation) may help prevent redistribution hypothermia 14,21. In contrast, during anaesthesia, peripheral cutaneous vasoconstriction contributes to reducing the decrease in core temperature by redistribution. Therefore, infusion of intraoperative vasoconstrictors (e.g. phenylephrine) may help prevent hypothermia 25. In addition, efforts should be made to minimize the time from the end of prewarming to the resumption of intraoperative active warming, and active interventions for the prevention of hypothermia, such as high ambient operating room temperature, use of warmed intravenous and irrigation fluid, active cutaneous warming, active airway heating and humidification, and minimisation of the exposed skin surface area, should be considered during surgery in elderly patients 1,2.

This study has some limitations. First, core temperature was measured using an infrared tympanic thermometer while the patient was awake. The reliability of an infrared thermometer is controversial because it commonly provides only the temperature of the external auditory canal or near the temporal artery 8. However, since the core temperature of the patient during anaesthesia was measured in the nasopharynx using a thermistor, the temperature measurement method would not have affected the primary outcome of this study and would have had little effect on secondary outcomes, except for changes in temperature during the perioperative period. Second, because of the group allocation according to age, there was a significant difference between the two groups in terms of weight and ASA PS. Low body weight and ASA PS > class I are risk factors for perioperative hypothermia 3. There is a need to conduct studies that match weight and ASA class to accurately evaluate the efficacy of prewarming according to age. Third, non-prewarmed patients were not included in this study. Therefore, effects according to the application of short-term prewarming in elderly patients could not be analyzed. In a previous study 18, the effect of prewarming for 5-15 min persisted until the first postoperative hour. Similarly, in our study, the temperature of elderly patients showed a tendency to gradually increase from 30 min to 60 min after arriving at PACU, which supports the importance of prewarming in elderly patients.

In conclusion, the effects of short-term prewarming for 20 min on the prevention of hypothermia and maintenance of perioperative normothermia are not the same in the elderly and adults. After 20 min of short-term prewarming, intraoperative hypothermia was more common, and the severity of hypothermia was worse in elderly patients than in adult patients. In PACU, the occurrence of postoperative shivering was more frequent in elderly patients, and the demand for active warming was also higher. Therefore, more careful thermal management should be considered and additional strategies to prevent inadvertent hypothermia are needed in elderly patients compared to adult patients.

## Author Contributions

All the listed authors were involved in the drafting of the work, approved the final manuscript, and agreed to be accountable for all aspects of this work.

***Sung-Ae Cho:*** This author helped in writing the manuscript and analyzing and interpreting data.

***Sieun Yoon, Seok-Jin Lee, Young-Seok Jee, Choon-Kyu Cho:*** These authors helped in the acquisition, analysis, and interpretation of data.

***Tae-Yun Sung:*** This author helped with the conception and design of the study, statistical analysis, and the writing of the manuscript.

## Figures and Tables

**Table 1:** Patient characteristics and perioperative data

Variable	Elderly (*n* = 24)	Adult (*n* = 25)	*P*
Age (years)	74 [67-84]	41 [38-47]	< 0.001
Sex (Male)	14 (58.3%)	11 (44%)	0.316
Weight (kg)	61.9 (13.6)	74.2 (14.3)	0.004
Height (cm)	155.6 (10.0)	166.1 (9.0)	< 0.001
Body mass index (kg.m^-2^)	25.4 (3.6)	26.8 (3.9)	0.206
ASA classification (II)	24 (100%)	17 (68%)	0.004
**Position of stone**			0.610
Ureter	12 (50.0%)	14 (56%)	
Kidney	8 (33.3%)	8 (32%)	
Both	4 (16.7%)	3 (12%)	
Preoperative core temperature (°C)	36.97 (0.30)	37.0 (0.27)	0.688
Preoperative thermal comfort score	8 [6-10]	8 [5.5-10]	0.878
Changed set temperature of prewarming from 43°C to 38°C	0 (0%)	1 (4.0%)	> 0.999
Cessation of prewarming to induction of anaesthesia (min)	8.6 (2.3)	7.6 (2.7)	0.149
Cessation of prewarming to start of intraoperative warming (min)	22.1 (3.1)	20.5 (2.6)	0.046
**Operating room temperature (°C)**			
Start of surgery	21.8 (1.1)	21.4 (1.1)	0.192
End of surgery	22.6 (1.2)	22.1 (1.5)	0.225
Intravenous fluid (ml)	325 [250-400]	250 [200-350]	0.141
Irrigation fluid (ml)	1350 [962.5-2225]	1000 [825-1750]	0.211
Estimated blood loss (ml)	3 [1-5]	2 [1-5]	0.248
Duration of surgery (min)	45 [40-63.8]	40 [32-72.5]	0.630
Duration of anaesthesia (min)	70 [60-85]	65 [55-95]	0.711

Data are presented as median [interquartile range], mean ± standard deviation, number, or number (%). Thermal comfort scale: 0=completely uncomfortable, 10=completely comfortable. ASA, American Society of Anesthesiologists.

**Table 2:** Perioperative outcomes during the study period

Variable	Elderly (*n* = 24)	Adult (*n* = 25)	Relative risk or Mean difference (95% confidence interval)	Effect size *d* or *h*	*P*
**In operating room**					
Incidence of hypothermia	14 (58.3%)	3 (12.0 %)	2.6 (1.5, 4.6)	1.030	0.001
**Severity of hypothermia**					0.002
Mild (35-35.9°C)	10 (41.7%)	3 (12.0%)	29.7% (4.8%, 50.7%)	0.697	
Moderate (34-34.9°C)	4 (17.7%)	0 (0%)	16.7% (0.02%, 35.9%)	0.868	
Severe (≤ 34°C)	0 (0%)	0 (0%)	Not applicable	0	
**In PACU**					
Numerical rating scale for pain	0 (0-0)	0 (0-3)	Not applicable	0.423	0.057
Fentanyl use	2 (8.3%)	5 (20%)	-11.7% (-31.7%, 9.1%)	0.343	0.417
Thermal comfort score	8 (3-10)	9 (6-10)	Not applicable	0.412	0.264
Incidence of shivering	8 (33.3%)	2 (8.0%)	2.0 (1.2, 3.2)	0.657	0.037
**Shivering score**					
1	8 (33.3%)	2 (8.0%)	25.3% (2.5%, 46.1%)	0.657	0.037
2	0 (0%)	0 (0%)	Not applicable	0	> 0.999
3	0 (0%)	0 (0%)	Not applicable	0	> 0.999
Active warming required	16 (66.7%)	8 (32.2%)	34.7% (6.7%, 56.0%)	0.705	0.015

PACU: post-anaesthesia care; NRS: numerical rating scale ranging from 0=no pain to 10=worst possible pain; thermal comfort scale: 0=completely uncomfortable to 10=completely comfortable; shivering score: 0=no shivering; 1=intermittent, low-intensity shivering; 2=moderate shivering; 3=continuous, intense shivering.

**Table 3:** Pairwise comparison of perioperative core temperature (°C) between groups at each assessment time

Time	Elderly		Adult		Mean difference (95% confidence interval)	*P*
	Temperature	*n*	Temperature	*n*		
Baseline	36.97 (0.31)	24	37.0 (0.26)	25	-0.03 (-0.25 - 0.18)	0.758
Before GA	36.9 (0.39)	24	37.10 (0.34)	25	-0.12(-0.34 - 0.09)	0.265
GA 0	36.30 (0.39)	24	36.55 (0.30)	25	-0.25 (-0.46 - -0.03)	0.024
GA 15	36.27 (0.39)	24	36.46 (0.24)	25	-0.19 (-0.4 - 0.02)	0.076
GA 30	36.13 (0.43)	24	36.36 (0.22)	25	-0.24 (-0.45 - -0.02)	0.032
GA 45	35.96 (0.42)	24	36.27 (0.25)	25	-0.31 (-0.53 - -0.10)	0.004
GA 60	35.93 (0.47)	13	36.28 (0.34)	9	-0.35 (-0.61 - -0.09)	0.009
GA 75	35.81 (0.44)	5	36.20 (0.27)	9	-0.38 (-0.69 - -0.08)	0.015
PACU 0	35.92 (0.54)	24	36.30 (0.31)	25	-0.38 (-0.60 - -0.17)	0.014
PACU 15	35.85 (0.53)	24	36.26 (0.31)	25	-0.42 (-0.62 - -0.20)	< 0.001
PACU 30	35.91 (0.50)	24	36.34 (0.32)	25	-0.43 (-0.63 - -0.22)	< 0.001
PACU 45	36.03 (0.53)	24	36.40 (0.33)	25	-0.37 (-0.58 - -0.15)	< 0.001
PACU 60	36.16 (0.47)	24	36.41 (0.27)	25	-0.25 (-0.46 - -0.04)	0.023

The temperature was estimated using a linear mixed model. Values are presented as the mean ± standard deviation. *p*-value was corrected using the Bonferroni method. Baseline, temperature at the arrival of the pre-operative holding area; Before GA, temperature on arrival in the operating room; GA 0-GA 60, temperature immediately after 60 min of induction of general anaesthesia (checked every 15 min), PACU 0-PACU 60: temperature immediately after 60 min of arrival in the post-anaesthetic care unit (checked every 15 min).

**Table 4:** Adverse events

Variable	Elderly (*n* = 24)	Adult (*n* = 25)	*p*-value
CRBD	7 (29.2%)	6 (24%)	0.682
Sore throat	4 (16.7%)	4 (16%)	> 0.999
Nausea	1 (4.2%)	3 (12%)	0.609
Sputum	1 (4.2)	1 (4%)	> 0.999
Vomiting	0 (0%)	1 (4%)	> 0.999
Dyspnoea	0 (0%)	1 (4%)	> 0.999
Dizziness	0 (0%)	1 (4%)	> 0.999

CRBD, catheter-related bladder discomfort.
